# HGDiscovery: An online tool providing functional and phenotypic information on novel variants of homogentisate 1,2- dioxigenase

**DOI:** 10.1016/j.crstbi.2022.08.001

**Published:** 2022-08-30

**Authors:** Malancha Karmakar, Vittoria Cicaloni, Carlos H.M. Rodrigues, Ottavia Spiga, Annalisa Santucci, David B. Ascher

**Affiliations:** a Computational Biology and Clinical Informatics, Baker Heart and Diabetes Institute, Melbourne, Victoria, Australia; b Systems and Computational Biology, Bio21 Institute, University of Melbourne, Melbourne, Victoria, Australia; c Department of Biotechnology, Chemistry and Pharmacy, University of Siena, Siena, Italy; d School of Chemistry and Molecular Biology, University of Queensland, Brisbane, Queensland, Australia

**Keywords:** Alkaptonuria, Structural bioinformatics, Machine learning, Precision medicine, Rare genetic disorder

## Abstract

Alkaptonuria (AKU), a rare genetic disorder, is characterized by the accumulation of homogentisic acid (HGA) in the body. Affected individuals lack functional levels of an enzyme required to breakdown HGA. Mutations in the homogentisate 1,2-dioxygenase (HGD) gene cause AKU and they are responsible for deficient levels of functional HGD, which, in turn, leads to excess levels of HGA. Although HGA is rapidly cleared from the body by the kidneys, in the long term it starts accumulating in various tissues, especially cartilage. Over time (rarely before adulthood), it eventually changes the color of affected tissue to slate blue or black. Here we report a comprehensive mutation analysis of 111 pathogenic and 190 non-pathogenic HGD missense mutations using protein structural information. Using our comprehensive suite of graph-based signature methods, mCSM complemented with sequence-based tools, we studied the functional and molecular consequences of each mutation on protein stability, interaction and evolutionary conservation. The scores generated from the structure and sequence-based tools were used to train a supervised machine learning algorithm with 89% accuracy. The empirical classifier was used to generate the variant phenotype for novel HGD missense mutations. All this information is deployed as a user friendly freely available web server called HGDiscovery (https://biosig.lab.uq.edu.au/hgdiscovery/).

## Highlights

• Functional and phenotypic consequences of HGD non-synonymous variations.
• Biophysical, structural and evolutionary analysis of novel and known clinical variants.
• Pathogenic mutations affected protein stability and conformational flexibility.
• Pathogenic mutations associated with deleterious scores for sequence-based features.
• HGDiscovery (http://biosig.unimelb.edu.au/hgdiscovery/) – webserver.

## Graphical abstract

**Figure:**
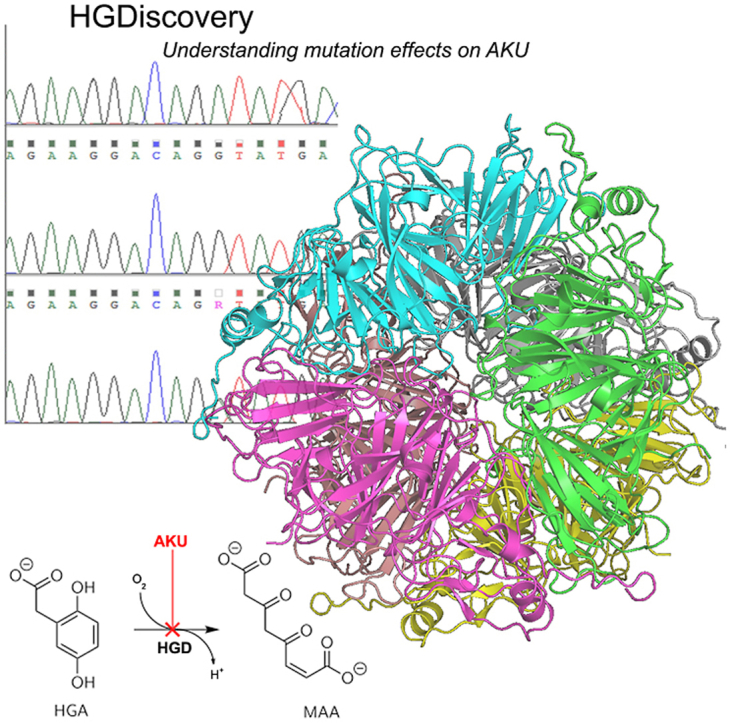

## 1 **Introduction**

Alkaptonuria (AKU) is a rare recessive metabolic disorder which was used by Sir Archibald Garrod in his Croonian lectures to describe one of the inborn errors of metabolism (Garrod, 2002). It is a hereditary disorder, resulting from mutations in the gene encoding the enzyme homogentisate 1,2 dioxygenase (HGD) (EC 1.13.11.5), responsible for the breakdown of homogentisic acid (HGA) which is an intermediate metabolite in the tyrosine degradation pathway (Phornphutkul et al., 2002). With blockage in tyrosine metabolism, elevated levels of HGA leads to deposition of its own polymers as an ochronotic pigment in the connective tissue including cartilage, heart valves, and sclera (Damarla et al., 2017). Manifestation of disease during early childhood is seen as “homogentisic aciduria”, which is darkening of the urine upon standing. Delayed symptoms can be seen after 30 years of age which involve “ochronosis” – pigmentation of collagenous tissues like cardiac valves, eyes, ears and skin (Zatkova et al., 2020). Current estimates of the disease occurrence in the Unites States obtained from the National Organization of Rare Disorders is 1 in 250,000–1,000,000 live births (Disorders, 2019).

HGD gene located on chromosome 3q21-q23 (Pollak et al., 1993). It is a single copy gene composed of 14 exons (Fernandez-Canon et al., 1996). Due to compound heterozygosity or homozygosity of HGD gene variants, the enzymatic defect in HGD is autosomal recessive (Pollak et al., 1993; Janocha et al., 1994). Information on all variants identified till date have been globally documented in the HGD mutation database (http://hgddatabase.cvtisr.sk/).

The experimental crystal structure of the HGD protein has been solved (PDB code 1EY2 and 1EYB) in 2000. The HGD protein protomer (NP_000178.2), is composed of 445 amino acids, which includes a 280 residue N-terminal domain, a central β-sandwich and a 140 residue C-terminal domain (Janocha et al., 1994). It is a complex hexameric protein arranged as a dimer of trimers (Titus et al., 2000). It is principally expressed in osteoarticular compartment cells (i.e. chondrocytes, synoviocytes and osteoblasts) (Laschi et al., 2012) and in prostate, small intestine, colon, kidney and liver (Fernandez-Canon et al., 1996). The spatial structure of the protomer, two-disc like trimers and the hexamer are maintained by an intricate network of non-covalent inter and intra-molecular interaction. This makes the protein structure extremely vulnerable to mutations (Nemethova et al., 2016).

Understanding the clinical impact of a rare genetic variants is a preeminent challenge in human genetics. Accurate predictions of variant's impact are an essential step towards precision medicine. The major obstacle in studying an ultra-rare and complex disease like AKU is the lack of a standardized methodology to assess disease severity and response to treatment (Ranganath and Cox, 2011), which is complicated by the fact that AKU symptoms differ from one individual to another. Detailed evaluation and comparison of clinical and genomic data of AKU patient can play a key role to understand AKU variability. An in-depth molecular characterization of the disease is needed in pharmacogenomics prediction for suitable medical treatment. To address the issue, ApreciseKUre platform was developed, which includes data on potential biomarkers, patients' quality of life, biochemical outcomes and clinical information facilitating their integration and analysis in order to shed light on pathological characterization of every AKU patient in a typical precision medicine perspective (Cicaloni et al., 2019; Spiga et al., 2017, 2018, 2020).

A further extension of the above method is proposed in this paper, where we describe a new database which would complement the existing ApreciseKUre database. The new database would provide the necessary underlying molecular information for novel and known clinical HGD variants. Structure and sequence-based information has been used to build a predictive tool using supervised machine learning algorithm. The tool has been implemented through the webserver HGDiscovery (https://biosig.lab.uq.edu.au/hgdiscovery/), providing functional and phenotypic information on non-synonymous HGD variations to guide clinical decisions. Moreover, HGDiscovery has a higher performance compared to the existing generic genetic tools designed for missense variants predictions such as SIFT (Sim et al., 2012), PMut (López-Ferrando et al., 2017) and PolyPhen 2 (Adzhubei et al., 2010).

## 2 Methods

### 2.1 Data curation

After removal of duplicate mutations, we curated a dataset composed of 301 non-synonymous substitutions. It included 190 non-pathogenic non-synonymous variations retrieved from gnomAD v.3 (Genome build GRCh38/hg38, Ensembl gene ID: ENSG00000113924.11, Region 3:120628173–120682571) (Lek et al., 2016) and 111 AKU-causing clinical mutations. The 111 variants were first described in the study of Ascher et al., 2019) (Ascher et al., 2019) and included in HGD Mutation Database (http://hgddatabase.cvtisr.sk) (Zatkova et al., 2012), which summarizes results of mutation analysis from approximately 530 AKU patients reported so far.

### 2.2 HGD protein structure

The X-ray crystallographic 3D structure of *Homo sapiens* holo-HGD (holo-HGDHs, PDB ID: 1EY2) is incomplete; thus, it needed structural reconstruction of the missing residues of the monomer and then of the whole hexamer in order to be able to perform a complete evaluation of variants effect on protein stability and flexibility. The missing loop in the human protein structure (residues 348–355) was reconstructed by homology modeling using the *Pseudomonas putida* HGD (HGDPp) structure. By using protein BLAST (Altschul et al., 1990) software three structures belonging to *Pseudomonas putida* were found, with a sequence identity larger than 49% and a root-mean-square deviation amounting to 1.8 ​Å for Cα (Jeoung et al., 2013). We opted for HGDPp, with PDB ID 4AQ2 since, similar to 1EY2, it has no substrate. The structures of holo-HGDHs (PDB ID: 1EY2) and its homologous HGDPp (PDB ID: 4AQ2) were retrieved from the Protein Data Bank (PDB) (Berman et al., 2000). Sequence alignment of 1EY2 and 4AQ2 were performed on BLAST web server (Altschul et al., 1990), to model the missing residues. The modelling of the loop 348–355 was carried out using a homology model approach in which an elucidated structure of HGDPp loop was employed as template to model the structure of the protein of interest. The completed monomer structure served as a starting point for the reconstruction of the whole HGDHs oligomeric protein on the template of the asymmetric units of PDB entry 1EY2. The structure reliability was validated using PROCHECK (Laskowski et al., 1993). Additionally, the energy minimization of the hexameric protein was performed using GROMACS 5.0.2 (Berendsen et al., 1995) (for additional information see Supplementary Methods in (Ascher et al., 2019)).

### 2.3 Biophysical and evolutionary score generation

A thorough structural and sequence-based assessment was performed for all the HGD variants to account for the potential effects of AKU-causing mutations. Variations in protein-protein interactions between the different monomers of the hexamer HGD upon mutation was determined using mCSM-PPI2 (Rodrigues et al., 2019). Changes in protein stability and folding were determined using our in-house tools like mCSM-Stability (Pires et al., 2014a), SDM (Pandurangan et al., 2017) and DUET (Pires et al., 2014b); and conformational flexibility changes using the normal mode analysis tool called DynaMut (Rodrigues et al., 2018). Effects of mutations on binding affinity of HGD to its substrate homogentisic acid were analysed using mCSM-Lig (Pires et al., 2016). All these are novel machine learning approaches that use graph-based signatures to represent the structural and biochemical environment of the wild-type 3D structure of a protein to quantitatively predict the effects of point mutation. To complement the structure-based methods, sequence-based feature like SNAP2 (Screening for Non-Acceptable Polymorphisms) (Hecht et al., 2015), ConSurf (Ashkenazy et al., 2016) and Provean (Protein Variation Effect Analyzer) (Choi and Chan, 2015) were used, which provides valuable evolutionary information. To further enrich the analysis, protein's wild type structural information such as residue depth, dihedral angles of the HGD chain φ (phi) and ψ (psi), relative solvent accessibility and secondary structure information were included. Changes in molecular interactions such as hydrophobic, ionic, van der Waals', hydrogen bonds and π interactions (cation–π, donor–π, carbon–π, π–π) between the wild type and mutant structures were calculated using Arpeggio (Jubb et al., 2017). Population-based variability was included using the missense tolerance ratio (MTR) (Traynelis et al., 2017) scoring system.

### 2.4 Supervised machine learning for empirical model building

The Scikit-learn Python library was used to evaluate different supervised machine learning algorithms for classification. These include – K-Nearest Neighbors (KNN), Random Forest, Decision Trees, Extra Trees, AdaBoost, Gradient Boosting, SVM, Gaussian Naïve Bayes, and Stochastic Gradient Descent. The best performing model was chosen by assessing metrics like Matthews correlation co-efficient (MCC), Area Under the Receiver Operating Characteristic (AUROC) curve, accuracy, F1-score and precision. The model was trained using stratified 10-fold cross validation. We carefully split the training and blind test dataset non-redundantly with respect to the amino acid residue position.

To address the issue of imbalance between the pathogenic and non-pathogenic mutations in the data, we evaluated the model performance by both under-sampling the non-pathogenic mutations and oversampling pathogenic mutations in the training dataset (Krawczyk, 2016). The performance was compared for above mentioned scenario with the normal dataset and best results were obtained when the pathogenic mutations were oversampled using the Extra Tree algorithm. **Ex**tremely **ra**ndomized **tree** classifier (or Extra Tree) is an ensemble machine learning algorithm and a variation of the random forest algorithm. The empirical binary classifier built using this algorithm highlights a set of structural and evolutionary features which can be used to discriminate between AKU-causing and non-pathogenic variations.

### 2.5 Webserver development

We have implemented HGDiscovery as a user-friendly and freely available webserver (http://biosig.unimelb.edu.au/hgdiscovery/). The front-end of the server was developed using Materialize css framework version 1.0.0, while the back-end was built in Python using the Flask framework version 1.0.2. The server is hosted on a Linux server running Apache 2.

## 3 Results

**Fig. 1:**
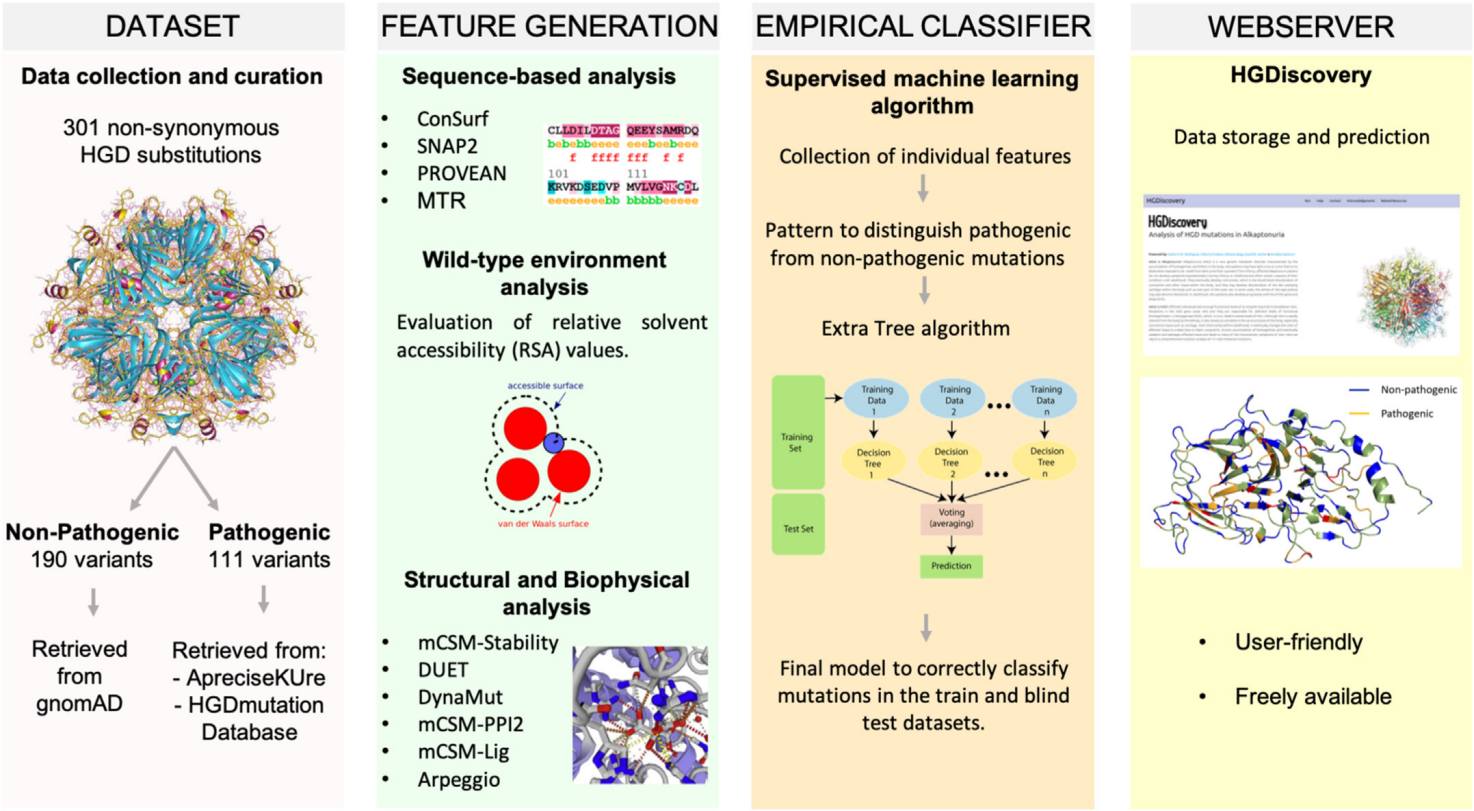
HGDiscovery workflow. The first step involves scoping published literature and clinical databases to prepare a curated list of non-synonymous HGD mutations. The second step involves generating various structure and sequence-based features for the curated missense mutations. In the third step, we use these features in a supervised machine learning algorithm to build a binary classifier, which can distinguish between pathogenic and non-pathogenic missense mutations. Finally, we develop a free available user-friendly webserver which contains phenotypic information on all HGD variants.

In this work, the 3D protein structure was used to understand the functional and molecular consequences of mutations in HGD leading to AKU disease and using the information generated from this analysis, a supervised machine learning algorithm was trained to develop a predictive tool to determine novel variants which could lead to AKU manifestation. Fig. 1 depicts the novel methodological pipeline developed.

### 3.1 Structural and biophysical analysis

**Fig. 2:**
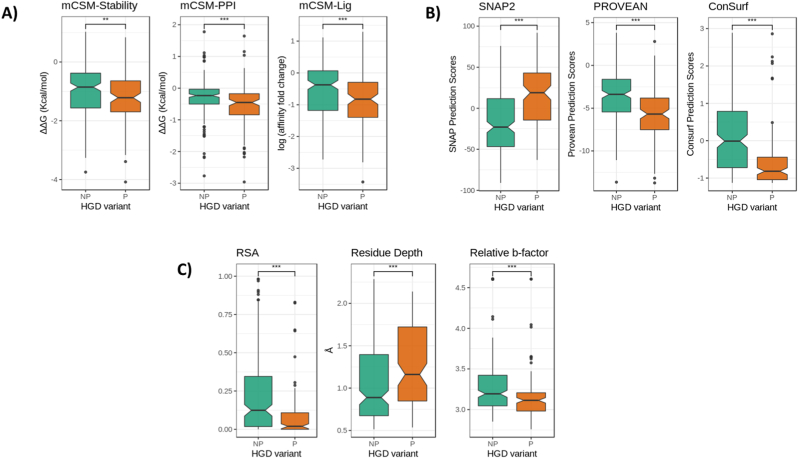
Boxplot representation of features. A) Structural features. B) Sequence based features. C) Wild-type environment features. The non-pathogenic mutations (NP) are represented as sea green and pathogenic mutations (P) as dark orange. (∗∗∗p ​< ​0.0001, ∗∗p ​< ​0.001, Welch two sample *t*-test). (For interpretation of the references to color in this figure legend, the reader is referred to the Web version of this article.)

Our in-house biophysical tools mCSM-Stability (Pires et al., 2014a), DUET (Pires et al., 2014b) and DynaMut (Rodrigues et al., 2018) were used to study and understand the impact of missense mutations on protein stability, folding and conformational flexibility. These tools are novel machine-learning algorithms which rely on graph-based signatures to calculate changes in Gibb's free energy upon non-synonymous mutations. We observed pathogenic mutations to be associated with highly destabilizing scores affecting protein stability and dynamics. The effects of mutation on the substrate binding affinity to active site were determined using mCSM-Lig (Pires et al., 2016). Pathogenic mutations altered the active/substrate binding pocket. mCSM-PPI2 (Rodrigues et al., 2019) was used to assess changes in protein-protein interaction and we observed pathogenic mutations hindered the formation of the symmetrical homo-hexamer. Therefore, pathogenic mutations either reduced or disrupted the HGD protein activity as seen in Fig. 2A.

### 3.2 Sequence-based analysis of HGD variants

ConSurf, SNAP2 and PROVEAN are sequence-based predictors and consider evolutionary information to predict functionally important non-synonymous mutation. The prediction helps us to understand the biological impact of a mutation on the protein structure. A consistent pattern was observed from all of the sequence-based features (Fig. 2B). The pathogenic mutations were associated with deleterious scores and the non-pathogenic mutations scored neutral. All the features were sufficiently statistically significant to be used to train the predictive algorithm to build the empirical tool (p-values SNAP2: 4.6 e^−14^, PROVEAN: 1.1 e^−9^, ConSurf: 2.4 e^−10^). Population-based variability was considered using the missense tolerance ratio (MTR) scoring system. Majority of the pathogenic mutations were in the bottom 25th percentile, reflecting intolerance and hence associated with altering protein function.

### 3.3 Wild-type structural environment analysis

The wild-type environment analysis (Fig. 2C) includes data on relative solvent accessibility (RSA), residue depth, dihedral angles and secondary structure information for both pathogenic and non-pathogenic variants. Looking into the relative solvent accessibility values for the pathogenic and non-pathogenic mutations (p-value: 2.2 e^−8^), we see pathogenic mutations tend to be less exposed than non-pathogenic variants. It has been previously described that the HGD protomer structure constitutes of a pore in which the side chains of large number of residues are exposed (Jeoung et al., 2013). These residues are thought to play an important part in the complex HGD catalytic function, and we see subtle changes in the side chains as non-synonymous substitution can affect the active site functionality (Ascher et al., 2019). The residue depth values reveal pathogenic mutations are more buried than non-pathogenic mutations. This observation is congruous with earlier observation where point mutations on the surface were better tolerated in the globular hexameric HGD protein structure.

### 3.4 Supervised machine learning algorithm: Extra Tree

**Fig. 3:**
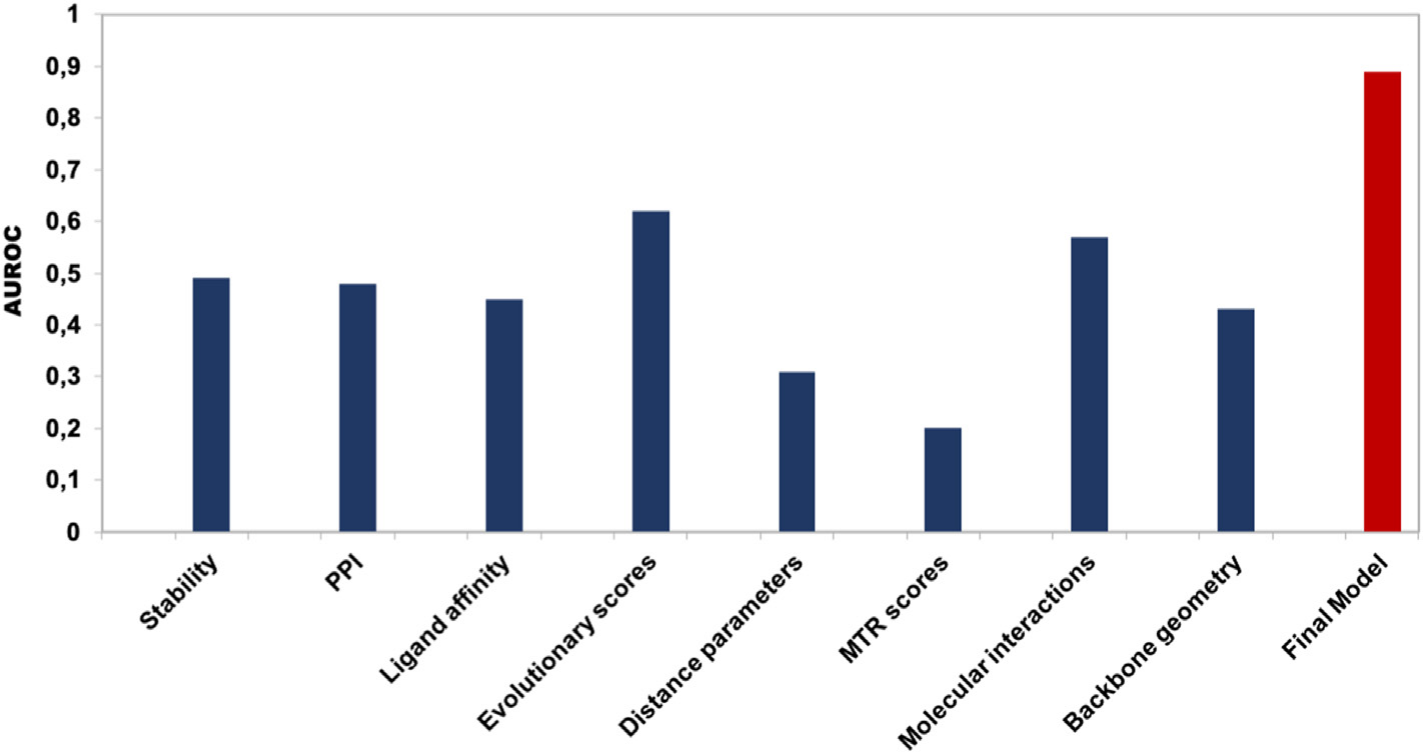
Empirical model performance trained on individual class of features. The Extra Tree algorithm was trained using stratified 10-fold cross validation using eight distinct class of features (first 8 ​bars from left to right; dark blue bars) and with a combination of all features (red bar). The AUROC score is low when a single class of feature is used for training the binary classifier, however, a significant improvement is noticed when all the eight different features are combined to build the model. (For interpretation of the references to color in this figure legend, the reader is referred to the Web version of this article.)

Our features could be grouped into eight distinct categories – protein stability, protein-protein interactions, ligand affinity, evolutionary conservation scores, distance parameters, MTR scores, molecular interaction and backbone geometry. Each category of features was initially used to build and evaluate the performance of the predictive model. After a thorough analysis of the individual features, a model was built using all eight distinct categories of features. A robust and balanced performance was observed when features were combined together (Table 1, Fig. 3).

**Table 1:** Performance metrics for the training and blind test dataset.

Dataset	AUROC	MCC	Precision	Recall	F-score
Training	0.89	0.58	0.79	0.79	0.79
Blind test	0.79	0.65	0.86	0.78	0.79

**Fig. 4:**
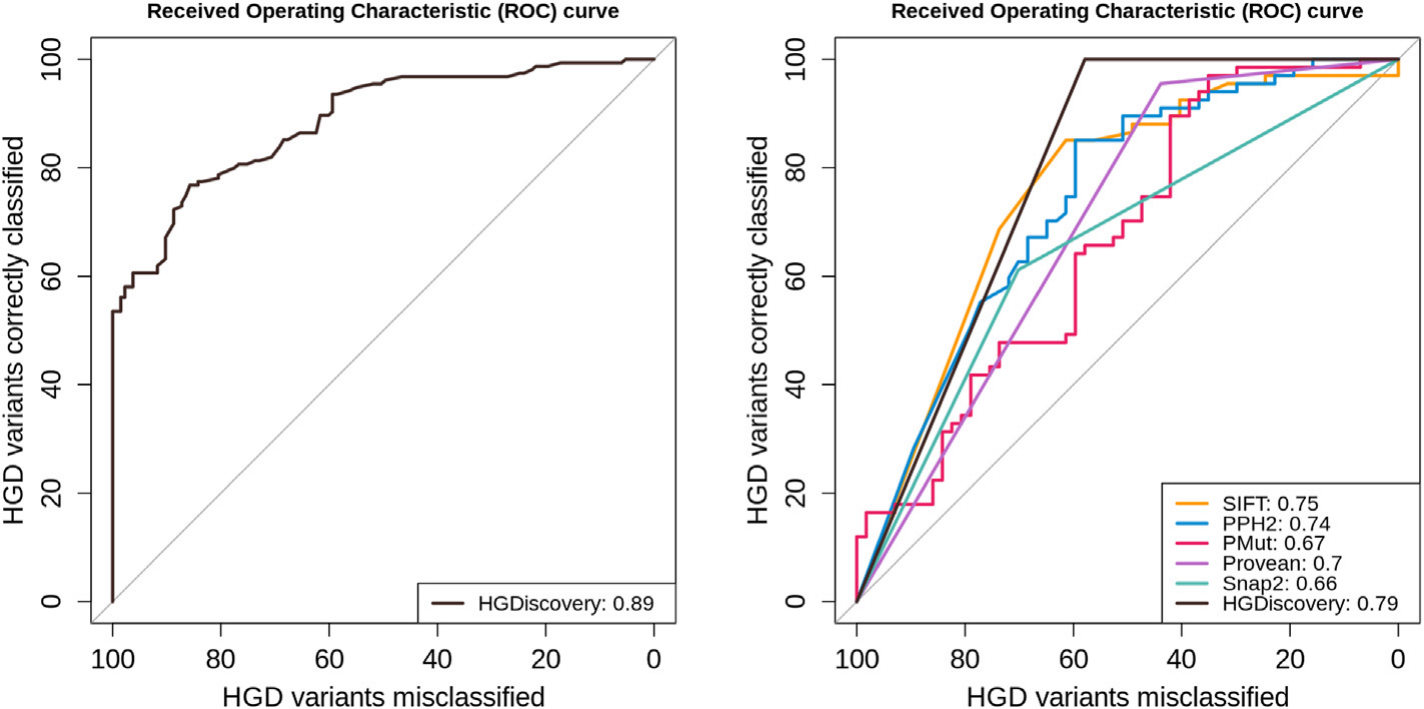
**Area Under the Receiver Operating Characteristic (AUCROC) curves of HGD classifier.** The AUROC shown for training and test datasets. The model is robust and outperforms the existing genetic tools like SIFT, PolyPhen 2 (PPH2), PMut.

190 non-pathogenic and 111 pathogenic mutations were split into non-redundant training and blind test datasets with respect to their amino acid position. Initially we observed poor performance on the model's ability to predict pathogenic mutation. We concluded that the training data set was imbalanced as there were more non-pathogenic mutations than pathogenic mutations. Oversampling (duplicating) (Krawczyk, 2016) the pathogenic mutations in the training dataset improved the metric scores. The final model correctly classified 89% and 79% of mutations in the training and blind test datasets respectively (Fig. 4).

Our model was then compared to the existing genetic tools like SIFT, PolyPhen 2 and PMut. The AUROC for SIFT, PolyPhen 2 and PMut were 0.75, 0.74 and 0.67 respectively. Comparison were also done against PROVEAN and Snap2 (generic missense variant predictors included in our predictive modeling). The score obtained for PROVEAN and Snap2 are 0.70 and 0.66 respectively. Therefore, HGDiscovery (AUROC 0.79) clearly outperforms the available methods which are not gene specific.

### 3.5 HGDiscovery webserver

HGDiscovery allows for users to query for a single point mutation or submit a list of mutations to be analysed in batch. For the “Single Mutation” option users are asked to provide the point mutation as a string containing the wild-type residue one-letter code, its corresponding residue number and the mutant residue one-letter code. The “Mutation List” option requires that a text file is submitted with the list of mutations (one per line).

The results page for the “Single Mutation” option displays the predicted outcome on the top alongside with details of the input mutation, wild-type residue environment, the variables and scores used by our predictive model and external links to experimental evidence (when available). An interactive 3D viewer using the NGL-viewer (Rose and Hildebrand, 2015) shows the molecular contacts generated by Arpeggio (Jubb et al., 2017) for wild-type and mutant structures.

On the “Mutation List” option, the results are displayed as a downloadable table. Individual analysis for each variant on the table can be analysed similarly to “Single Mutation” option by clicking the “Details” button. An interactive viewer is also shown at the bottom of the page highlighting Pathogenic and Non-pathogenic mutations on the 3D structure.

## 4 Discussion

Here we present an empirical classifier, HGDiscovery, which has phenotypic information on all known variants of homogentisate 1,2 dioxygenase, (EC 1.13.11.5), an enzyme involved in the metabolism of tyrosine, whose deficiency leads to Alkaptonuria [OMIM 203500]. Structural, evolutionary and molecular information from known HGD variants were combined to look for investigative patterns which could distinguish non-pathogenic from AKU-causing non-synonymous variants. So along with physiological information from ApreciseKUre platform, an additional AKU-dedicated database is available, which provides new insight into functional and phenotypic consequences of novel HGD non-synonymous variations, crucial for a genetic disease like AKU to support clinical decisions.

The 3D crystal structure of the HGD active form reveals a highly complex and dynamic hexameric organization comprising two disk-like trimers (Titus et al., 2000). An intricate network of noncovalent interactions is needed to maintain the spatial structure firstly of the protomer, the trimer and then the hexamer. This delicate structure presents a very low tolerance to mutations and can be easily disrupted mainly by missense variants compromising enzyme function. In case of HGD, missense variants represent approximately 65% of all known AKU substitutions (Zatkova et al., 2020; Nemethova et al., 2016; Zatkova, 2011) and 93 distinct amino acid residue positions within the structure are affected by the 111 AKU-causing missense changes. AKU-causing mutations appear to reduce or disrupt the HGD protein activity by destabilizing its structure and altering the active site/substrate binding pocket. Our results along with studies on evolutionary conservation revealed that AKU variants were mainly located at more conserved residue positions (Ascher et al., 2019) and, consequently, HGD missense changes can influence protein folding and stability or interactions with other protomers or substrate. Specifically, they can decrease stability of individual protomers, disrupt protomer–protomer interactions, or modify residues in the active-site region. Thus, when a novel HGD missense mutation is identified, it is important to distinguish causal AKU variants from non-pathogenic ones.

Classical predictors of missense variants include SIFT, SNAP2, PROVEAN and PolyPhen 2. These are machine learning algorithms which are trained using sequence and evolutionary information. Though these tools are powerful for predictions but they are not gene specific. On the other hand, HGDiscovery is an amalgamation of sequence and structure-based features specifically built to understand mutations in HGD. To avoid potential biases in the predictive model, we used high quality manually curated mutation data, and ensured that mutations used in training and testing were not used in the development of computational tools that were considered as features. The structure-based features like protein stability, protein-protein interactions and conformational flexibility complement the sequence-based features like ConSurf and PROVEAN and helps in generating an empirical classifier which is robust and generalizable, with minimal loss in performance between training and independent blind tests. Future work will hopefully elucidate whether misclassified mutations were a consequence of noise in the clinical data, accuracy of predictive tools used to capture molecular consequences, or a reflection that additional biology needs to be incorporated into the model.

It is not uncommon that AKU patients carry compound heterozygotes for two HGD gene variants. In such cases, the estimation of the role of each missense variant is not trivial, since the hexamer could be assembled with monomers all affected by the same variant (homo-oligomer) or by two different ones (heterooligomer) (Gallagher et al., 2016). Variants affecting two different regions could have additive destructive effect, on the contrary, the effects could partially compensate for those that belong to the same region. However, we do not have any tools able to evaluate such events (Ranganath and Cox, 2011). Compound heterozygosity cannot be interfered with our analysis, leading to misclassification of variants. This was the limitation of our study. But with increasing availability of genomic and clinical data after patient analysis in future, we can always update our tool and re-label the mislabeled non-synonymous variants.

The information available from the above study can be used to develop new treatment strategies, for example, use of small molecules. We know that a pathogenic mutation with destabilizing scores for stability and flexibility leading to reduced enzyme activity can be rescued partially or totally with the help of a small molecule and hence might decrease the severity of the disease (Ascher et al., 2019). Therefore, this framework represents an online tool that can be turned into a best practice model for rare diseases. We believe this is not limited to the study of AKU, but it represents a proof of principle study that could be applied to other rare diseases, allowing data management, analysis and interpretation. Previously, this novel methodological pipeline has been applied to understand and determine novel drug resistant mutations in tuberculosis (Karmakar et al., 2019, 2020) and even performed a real-time analysis (Karmakar et al., 2018) on tuberculosis patient. Similarly, HGDiscovery, a user friendly freely available tool, could serve as a great additional source of interrogative model which helps in understanding the protein structure and function to design tailored drugs and effective therapies including gene therapy.

## Funding

M.K and C.H.M.R were funded by Melbourne Research Scholarships. D.B.A. was funded by the Jack Brockhoff Foundation [JBF 4186, 2016]; and an Investigator Grant from the 10.13039/501100000925National Health and Medical Research Council of Australia [GNT1174405]. Supported in part by the Victorian Government's OIS Program.

## Data availability

The data underlying this article will be shared on reasonable request to the corresponding author.

## CRediT authorship contribution statement

**Malancha Karmakar:** Data curation, machine learning, data Formal analysis. **Vittoria Cicaloni:** Data curation, machine learning, data Formal analysis. **Carlos H.M. Rodrigues:** developed the HGDiscovery website and assisted with machine learning. **Ottavia Spiga:** assisted with clinical mutation curation. **Annalisa Santucci:** assisted with clinical mutation curation. **David B. Ascher:** designed, Supervision.

## Declaration of competing interest

The authors declare that they have no known competing financial interests or personal relationships that could have appeared to influence the work reported in this paper.

## References

[bib1] Adzhubei I.A. (2010). A method and server for predicting damaging missense mutations. Nat. Methods.

[bib2] Altschul S.F. (1990). Basic local alignment search tool. J. Mol. Biol..

[bib3] Ascher D.B. (2019). Homogentisate 1,2-dioxygenase (HGD) gene variants, their analysis and genotype-phenotype correlations in the largest cohort of patients with AKU. Eur. J. Hum. Genet..

[bib4] Ashkenazy H. (2016). ConSurf 2016: an improved methodology to estimate and visualize evolutionary conservation in macromolecules. Nucleic Acids Res..

[bib5] Berendsen H.J.C., van der Spoel D., van Drunen R. (1995). GROMACS: a message-passing parallel molecular dynamics implementation. Comput. Phys. Commun..

[bib6] Berman H.M. (2000). The protein Data Bank. Nucleic Acids Res..

[bib7] Choi Y., Chan A.P. (2015). PROVEAN web server: a tool to predict the functional effect of amino acid substitutions and indels. Bioinformatics.

[bib8] Cicaloni V. (2019). Interactive alkaptonuria database: investigating clinical data to improve patient care in a rare disease. Faseb. J..

[bib9] Damarla N. (2017). Alkaptonuria: a case report. Indian J. Ophthalmol..

[bib10] Disorders N.O.f.R. (2019). https://rarediseases.org/rare-diseases/alkaptonuria/.

[bib11] Fernandez-Canon J.M. (1996). The molecular basis of alkaptonuria. Nat. Genet..

[bib12] Gallagher J.A. (2016). Alkaptonuria: an example of a "fundamental disease"--A rare disease with important lessons for more common disorders. Semin. Cell Dev. Biol..

[bib13] Garrod A.E. (2002). The incidence of alkaptonuria: a study in chemical individuality. Yale J. Biol. Med..

[bib14] Hecht M., Bromberg Y., Rost B. (2015). Better prediction of functional effects for sequence variants. BMC Genom..

[bib15] Janocha S. (1994). The human gene for alkaptonuria (AKU) maps to chromosome 3q. Genomics.

[bib16] Jeoung J.-H. (2013). Visualizing the substrate-, superoxo-, alkylperoxo-, and product-bound states at the nonheme Fe(II) site of homogentisate dioxygenase. Proc. Natl. Acad. Sci. USA.

[bib17] Jubb H.C. (2017). Arpeggio: a web server for calculating and visualising interatomic interactions in protein structures. J. Mol. Biol..

[bib18] Karmakar M. (2018). Analysis of a novel pncA mutation for susceptibility to pyrazinamide therapy. Am. J. Respir. Crit. Care Med..

[bib19] Karmakar M. (2019). Empirical ways to identify novel Bedaquiline resistance mutations in AtpE. PLoS One.

[bib20] Karmakar M. (2020). Structure guided prediction of Pyrazinamide resistance mutations in pncA. Sci. Rep..

[bib21] Krawczyk B. (2016). Learning from imbalanced data: open challenges and future directions. Progr. Artif. Intell..

[bib22] Laschi M. (2012). Homogentisate 1,2 dioxygenase is expressed in human osteoarticular cells: implications in alkaptonuria. J. Cell. Physiol..

[bib23] Laskowski R. (1993). PROCHECK: a program to check the stereochemical quality of protein structures. J. Appl. Crystallogr..

[bib24] Lek M. (2016). Analysis of protein-coding genetic variation in 60,706 humans. Nature.

[bib25] López-Ferrando V. (2017). PMut: a web-based tool for the annotation of pathological variants on proteins, 2017 update. Nucleic Acids Res..

[bib26] Nemethova M. (2016). Twelve novel HGD gene variants identified in 99 alkaptonuria patients: focus on 'black bone disease' in Italy. Eur. J. Hum. Genet..

[bib27] Pandurangan A.P. (2017). SDM: a server for predicting effects of mutations on protein stability. Nucleic Acids Res..

[bib28] Phornphutkul C. (2002). Natural history of alkaptonuria. N. Engl. J. Med..

[bib29] Pires D.E.V., Ascher D.B., Blundell T.L. (2014). mCSM: predicting the effects of mutations in proteins using graph-based signatures. Bioinformatics.

[bib30] Pires D.E.V., Ascher D.B., Blundell T.L. (2014). DUET: a server for predicting effects of mutations on protein stability using an integrated computational approach. Nucleic Acids Res..

[bib31] Pires D.E., Blundell T.L., Ascher D.B. (2016). mCSM-lig: quantifying the effects of mutations on protein-small molecule affinity in genetic disease and emergence of drug resistance. Sci. Rep..

[bib32] Pollak M.R. (1993). Homozygosity mapping of the gene for alkaptonuria to chromosome 3q2. Nat. Genet..

[bib33] Ranganath L.R., Cox T.F. (2011). Natural history of alkaptonuria revisited: analyses based on scoring systems. J. Inherit. Metab. Dis..

[bib34] Rodrigues C.H.M., Pires D.E.V., Ascher D.B. (2018). DynaMut: predicting the impact of mutations on protein conformation, flexibility and stability. Nucleic Acids Res..

[bib35] Rodrigues C.H.M. (2019). mCSM-PPI2: predicting the effects of mutations on protein–protein interactions. Nucleic Acids Res..

[bib36] Rose A.S., Hildebrand P.W. (2015). NGL Viewer: a web application for molecular visualization. Nucleic Acids Res..

[bib37] Sim N.L. (2012). SIFT web server: predicting effects of amino acid substitutions on proteins. Nucleic Acids Res..

[bib38] Spiga O. (2017). ApreciseKUre: an approach of precision medicine in a rare disease. BMC Med. Inf. Decis. Making.

[bib39] Spiga O. (2018). A new integrated and interactive tool applicable to inborn errors of metabolism: application to alkaptonuria. Comput. Biol. Med..

[bib40] Spiga O. (2020). Machine learning application for development of a data-driven predictive model able to investigate quality of life scores in a rare disease. Orphanet J. Rare Dis..

[bib41] Titus G.P. (2000). Crystal structure of human homogentisate dioxygenase. Nat. Struct. Biol..

[bib42] Traynelis J. (2017). Optimizing genomic medicine in epilepsy through a gene-customized approach to missense variant interpretation. Genome Res..

[bib43] Zatkova A. (2011). An update on molecular genetics of Alkaptonuria (AKU). J. Inherit. Metab. Dis..

[bib44] Zatkova A. (2012). Identification of 11 novel homogentisate 1,2 dioxygenase variants in alkaptonuria patients and establishment of a novel LOVD-based HGD mutation database. JIMD Rep.

[bib45] Zatkova A., Ranganath L., Kadasi L. (2020). Alkaptonuria: current perspectives. Appl. Clin. Genet..

